# Trends in forensic DNA database: transnational exchange of DNA data

**DOI:** 10.1080/20961790.2019.1565651

**Published:** 2019-02-07

**Authors:** Aaron Opoku Amankwaa

**Affiliations:** Science and Justice Research Interest Group, School of Law, Northumbria University, Newcastle Upon Tyne, UK

**Keywords:** Forensic DNA exchange, Prüm, forensic DNA legislation, genetic privacy, proportionality, social security

## Abstract

The transnational exchange of forensic DNA data has become a modern trend in fighting cross-border crime, terrorism and illegal immigration. Forensic DNA data allow the police to identify, eliminate or link individuals associated with a crime. Additionally, different crime scenes can be linked via the DNA profile to identify serial offenders or determine crime patterns. Approaches to the transnational exchange of DNA data can be categorized into four: (1) creation of an international DNA database, (2) linked or networked national DNA databases, (3) request-based exchange of data, and (4) a combination of these. Most countries operate the combination system of data exchange. This paper briefly introduces the different approaches in the transnational sharing of forensic DNA data, the legislative and operational framework, pattern of data exchange and participating states, and policy challenges associated with data sharing. Generally, most DNA exchange systems are modelled as the European Union Prüm regime. This operates under two stages: hit/no-hit query and further information sharing. The scope of the data exchange is governed by individual national legislation that determines the type of information that can be shared and the national authority responsible for the system. Though DNA data exchange has been instrumental in resolving serious crimes such as gang and serial rape, and armed robbery, adequate information about their overall effectiveness and efficiency is lacking. Further, operational, legal and ethical challenges including issues of privacy and proportionality appear to limit the full potential of the DNA data exchange system.

## Introduction

With the increase in cross-border crime, the international exchange of intelligence for policing purposes has become very necessary. Forensic DNA data support police detective work through identification of suspects/victims, elimination of individuals from a criminal inquiry or association of individuals to crime [1]. The power of forensic DNA data has been enhanced through the establishment of DNA databases, allowing the identification of unknown offenders and serial offenders by linking different crimes. Current trends in DNA database include the expansion of national databases, innovative application of databases (e.g. familial searching [2], analysis of mobility of offenders and crime patterns [3,4]), harmonization of international and domestic DNA legislation and policies, and transnational exchange of DNA records. Whilst databases may serve public security interests, they pose a significant threat to the civil liberties of individuals. Existing literature indicates concerns about privacy (including “function creep” and misuse of data), lack of data on the overall effectiveness of databases and public education gap about DNA databases [5–10].

This review focuses on approaches to forensic DNA data exchange, which has become a common trend over the last 2 decades. The paper briefly introduces the legal and operational framework, pattern of data exchange and participating states, and policy challenges associated with data exchange. The goal of the review is to enhance public and stakeholder understanding of DNA database and inform opinions about appropriate policies for DNA databases and their uses, including the exchange of data.

## Transnational exchange systems

The transnational exchange of DNA data involves four main approaches: (1) international DNA databases, (2) linked national DNA databases, (3) request-based exchange of data, and (4) a combination of these [10–12]. As demonstrated in the next sections, the combination system of data exchange is employed by most countries engaged in the sharing of forensic information.

### International DNA databases

International DNA databases are either “global” or regional. An example of the global system is the INTERPOL DNA Gateway platform that was established in 2002 [12]. As of December 2017, more than 84 countries were participating in the INTERPOL DNA Database (IDD) with a holding of 173 000 DNA profiles [13]. According to a UK Home Office release in 2015, the estimated time taken to exchange DNA data through the INTERPOL process is approximately 143 days [14,15]. The specific reasons for this timescale are not reported. The UK is a participant of the IDD and exports a limited number of DNA profiles to the INTERPOL database [16]. The IDD holds DNA profiles from convicted individuals, suspects, missing persons and unidentified human remains and crime scenes. The IDD excludes personal information of data subjects, and the profiles are governed by the national laws of the submitting law enforcement agency. For example, profiles originating from the UK will be subject to the rules set out in the Protection of Freedoms Act 2012 (PoFA) [17]. This policy indicates that, overall, domestic laws dictate access and uses of data and the protection of the rights of IDD data subjects. According to INTERPOL [12,18], the database was instrumental in Project Pink Panther (2007–2016), where a group of individuals involved in transnational jewellery thefts were apprehended.

Regional international DNA databases for criminal investigations are limited. One well-established regional criminal intelligence and information database is the Europol Information System (EIS) which includes a DNA database containing profiles from European Union (EU) Member States [19]. The EIS was established in 2005 and stores information on serious international crime, convicted and suspected individuals and other information related to crime. Like the INTERPOL database, profiles stored on the EIS are subject to national laws of the submitting agency. Access to data stored on the EIS can be restricted by the submitting agency and may only allow access where a hit is obtained [19]. According to Europol [20], the EIS holds more than 147 096 data of persons as of 2017. The Europol Programming Document indicates the agency is considering partnership with the EU Prüm framework to increase the scope and capabilities of its DNA and biometric exchange system with third countries [20,21].

### Linked or networked national DNA databases

The EU Prüm arrangement is modelled as a network of separate national databases of member countries [22,23]. Austria, Belgium, France, Germany, Luxembourg, the Netherlands and Spain signed the Prüm Treaty on 27 May 2005. The arrangement was adopted into EU legislation in 2008, requiring all member states to create a database that can be accessed by other member countries. Council Decision 2008/615/JHA [22] and Council Decision 2008/616/JHA [23] provide the legal framework for the EU Prüm regime. The types of intelligence covered under Prüm include DNA, fingerprints and vehicle registration information. The DNA data exchange operates under two stages: hit/no-hit query and further information sharing (articles 3, 4 and 5 of Council Decision 2008/615/JHA [22]). In the first stage, DNA data from one country is automatically searched on the database of another country to identify any matches. If a match is obtained, the case progresses to the second stage where identifying information of the data subject is shared with the requesting country. Currently, more than 6 million subject profiles and 1 million crime scene profiles are available for exchange, excluding data from the UK [24]. Compared to the INTERPOL exchange process, it takes approximately 15 min to exchange data via Prüm [14]. The Prüm regime requires all EU states to establish national contact points (NCPs) to facilitate and manage the exchange of intelligence data (article 6 of Council Decision 2008/615/JHA [22]). The operation of the data exchange scheme is governed by national legislation that determines the powers of NCPs.

Within Europe, the largest national DNA database is the UK National DNA Database (NDNAD), holding >6 million subject profiles and >590 000 crime scene profiles [25]. The large size of the NDNAD is due to multiple factors. Firstly, it is the oldest national DNA database in the world. Between 2001 and 2013, the minimum threshold for indefinite inclusion on the NDNAD was an arrestee of a recordable offence [7]. This was in contrast to the legal system in most other EU countries, which restrict inclusion to mainly serious crime offenders and continuous retention to fixed periods [26]. Following the ruling in the *Case of* *S. and Marper v. the United Kingdom* [27] in 2008, the rules governing DNA data have been changed in UK. The PoFA rules currently require DNA data of unconvicted people to be deleted after investigations or proceedings, or after a short fixed period (2–3 years) depending on the seriousness of offence [17]. Data from convicted individuals are subject to indefinite retention except some juveniles convicted of a first minor offence. The new law requires all DNA samples to be destroyed after a profile has been generated or within 6 months. Although these changes led to the destruction of a considerable number of DNA records, the NDNAD still holds more profiles than other EU states [28].

In December 2015, the UK opted to participate in the Prüm regime and inquiries into EU–UK security cooperation indicates strong support to retain the scheme post-Brexit [29–32]. A major limiting factor for UK participation in Prüm has been variations in legislation. Available policy guidance have set out specific conditions for UK participation including the restriction of searching to data of convicted individuals, crime scene profiles and unidentified human remains, and the establishment of an independent Prüm Oversight Board [29,33–35]. The UK Biometrics Commissioner reports that arrangements have been made to commence data exchange in 2018 [9]. This will significantly increase the number of profiles available for exchange through Prüm. As part of the DNA exchange arrangement, the Combined DNA Index System (CODIS) platform owned by the Metropolitan Police Service (MPS) will be used to facilitate data exchange.

According to the Council of the EU [35], as of September 2017, 24 member states were actively exchanging DNA data with other member states under the Prüm regime. This number had not increased at the end of June 2018 [21]. The non-operational states are Croatia, Ireland, Italy and the UK. Eight states were exchanging DNA data with 19–22 other states: the Netherlands (22), Germany (20), Austria (20), Slovakia (20), Slovenia (19), Romania (19), Hungary (19) and France (19). Five states were connected to less than 10 other states: Luxembourg (9), Bulgaria (8), Belgium (4), Denmark (1) and Greece (receiving support from the Netherlands). The remaining states are connected to 13–18 other states.

A study by Santos and Machado [36] analysed match data of operational states from 2011 to 2015. The study utilized “unfiltered” match statistics from the Prüm exchanges. A match is obtained where two independent profiles are the same or similar. The match can be a confirmed one or not. The confirmed match is where an initial hit is further tested to establish whether it is authentic or a false positive. The unfiltered matches are the initial hits from the database and include false positives. This limitation means that the findings from the study should be interpreted cautiously.

Santos and Machado [36] found that five early signatories of the initial Prüm Treaty consistently ranked high in the number of unfiltered matches from 2011 to 2015: Austria (1 463–2 989), France (737–5 666), Germany (4 532–7 068), the Netherlands (646–1 707) and Spain (908–2 443). The study assessed the pattern of exchange by computing the ratio of matches between a state’s national database and external crime stain (OP-ES) to match between outbound crime stain and external national database (OS-EP). A high ratio shows that a state’s national database is potentially more beneficial to other states than the benefit it derives from external national databases. In 2015, Romania (7.47), Lithuania (4.71) and Hungary (4.46) recorded the highest OP-ES/OS-EP. Overall, the study showed a disproportionate impact of member states in the transnational exchange of DNA data under the Prüm regime. Factors contributing to this observation include differences in legislation, size and age of databases, variation in operational procedures, criminal mobility patterns and uneven connection between states. To assess the effectiveness of the Prüm regime, the study recommended a follow-up of confirmed DNA hits through the criminal justice process. Secondly, to improve transparency, accountability and trust in the system, the study highlighted the need to assess stage 2 of the Prüm regime. Previous studies [37,38] have also made similar recommendations to ensure the sustainability of the transnational exchange of DNA data.

### Request-based exchange of DNA data

The request-based scheme of exchanging DNA data is practiced by several countries around the world. Countries with bilateral agreements allow conditional automated searching of databases for public security reasons. Features of this scheme include the requirement that the exchange of DNA information must be “necessary”, “relevant” and “proportionate” for a policing purpose and the prioritization of serious crimes [39]. Generally, the sharing of DNA data follows the two-stage process of Prüm. In the UK, the National Crime Agency (NCA) manages the international exchange of DNA data under this model [34]. The INTERPOL I-24/7 network is used as a channel for sharing data. One major disadvantage of this approach is the time taken to share information. The process has been described as time consuming compared to other approaches [24,40]. The small number of profiles exchanged under this approach may reflect this challenge.

According to the UK Biometrics Commissioner’s 2017 report [9], 23 subject profiles (including 16 profiles of missing persons) and 166 crime scene profiles (including 14 profiles of unidentified human remains) were sent from the UK from January to December 2017. Of the searches completed in foreign databases, the subject profiles yielded one positive or potential match, whilst the crime scene profiles yielded 13 matches. Within the same period, 107 subject profiles and 619 crime scene profiles were sent to the UK. The respective matches were nine for the subject profiles and 34 for the crime scene profiles. Contrary to UK policy on international DNA exchange [39], the 2016 report of the Biometrics Commissioner indicates instances where data of subjects have been exchanged with associated personal information at the first stage [34]. A second issue identified was the searching of DNA data related to offences other than qualifying (serious) offences [9,34]. Thirdly, there were instances where NDNAD searches were conducted without the approval of the Database Strategy Board [9,34]. The Biometrics Commissioner notes that measures have been implemented to address these issues and prevent future occurrence.

Like the UK, the request-based system is practiced through bilateral agreements by the US with Argentina, Australia, Austria, Bulgaria, Chile, Croatia, Cyprus, Czech Republic, Denmark, Estonia, Finland, France, Germany, Greece, Hungary, Iceland, Ireland, Italy, Latvia, Liechtenstein, Lithuania, Malta, New Zealand, Portugal, Romania, Slovenia, Slovakia, Sweden, the Netherlands and other states [41–67]. These bilateral agreements are modelled as the EU Prüm regime. However, there is a focus on serious crime and parties exercise autonomy in permitting automated searches of databases based on the principle of reciprocity. The extent of implementation of the US bilateral agreements for DNA data exchange is not clear [40].

## Conclusion

Over the past 2 decades, the fight against cross-border crime, terrorism and illegal immigration has intensified interest in the transnational exchange of forensic DNA data. It appears the largest exchange system is the EU Prüm framework, involving a network of multiple national DNA databases. There is a possibility for non-EU national (such as Norway, Iceland, Switzerland and Liechtenstein [21]), international or regional law enforcement agencies to partner the Prüm framework. This potential global network of databases may introduce significant “administrative burdens” on national database managers. Further, due to the volume of exchanges, databases may encounter difficulties in managing searches and false-positive matches. These challenges imply a need to develop strong algorithms for comparison as well as an expansion of existing standard set loci to increase the discriminatory power of profiles.

The review identified a common policy in all the available systems: the governance of data by domestic legislation and implementation of the two-stage Prüm process. Several studies have noted that national differences in operational, legal and ethical policies including privacy safeguards and interpretation of proportionality appear to limit the full potential of the DNA data exchange systems [37,38,68–71]. The current trend dictates a need for legal and operational harmonisation of domestic policies to protect both public security and individual civil liberties.

Whilst the utility of the DNA exchange system has been demonstrated in resolving serious crimes such as gang and serial rape, murder and armed robbery [12,13,18,24], there is limited information on the overall effectiveness and efficiency of this crime-fighting tool [36,72]. This knowledge base is critical to help establish whether the creation and operation of DNA exchange systems is “a good return on investment”.
